# High-Performance Face Milling of 42CrMo4 Steel: Influence of Entering Angle on the Measured Surface Roughness, Cutting Force and Vibration Amplitude

**DOI:** 10.3390/ma14092196

**Published:** 2021-04-25

**Authors:** Marcin Płodzień, Łukasz Żyłka, Paweł Sułkowicz, Krzysztof Żak, Szymon Wojciechowski

**Affiliations:** 1 Department of Manufacturing Techniques and Automation, The Faculty of Mechanical Engineering and Aeronautics, Rzeszow University of Technology, ul. W. Pola 2, 35-959 Rzeszow, Poland; plodzien@prz.edu.pl (M.P.); zylka@prz.edu.pl (Ł.Ż.); sulkowicz@prz.edu.pl (P.S.); 2 Department of Manufacturing and Materials Engineering, The Faculty of Mechanical Engineering, Opole University of Technology, 76 Proszkowska St., 45-758 Opole, Poland; k.zak@po.edu.pl; 3 Institute of Mechanical Technology, The Faculty of Mechanical Engineering, Poznan University of Technology, Piotrowo 3, 60-965 Poznan, Poland

**Keywords:** high feed milling, face milling, cutting force, vibration

## Abstract

High feed Milling is a new milling method, which allows to apply high feed rates and increase machining efficiency. The method utilizes face cutters with a very small entering angle, of about 10°–20°. Thus, the cut layer cross-section is different than in traditional milling. In order to examine the high feed milling (HFM), experimental tests were conducted, preceded by an analysis of cutting zones when milling with an HF face cutter. The face milling tests of 42CrMo4 steel with the use of an HF cutter characterized by an entering angle, dependent on axial depth of cut *a_p_* and insert radius *r* values, as well as with a conventional face cutter with the entering angle of 45° were performed. The study focused on analyzing the vibration amplitude, cutting force components in the workpiece coordinate system, and surface roughness. The experimental tests proved, that when milling with constant cut layer thickness, the high feed cutter allowed to obtain twice the cutting volume in comparison with the conventional face cutter. However, higher machining efficiency resulted in an increase in cutting force components. Furthermore, the results indicate significantly higher surface roughness and higher vibration amplitudes when milling with the HF cutter.

## 1. Introduction

**Figure 1:**
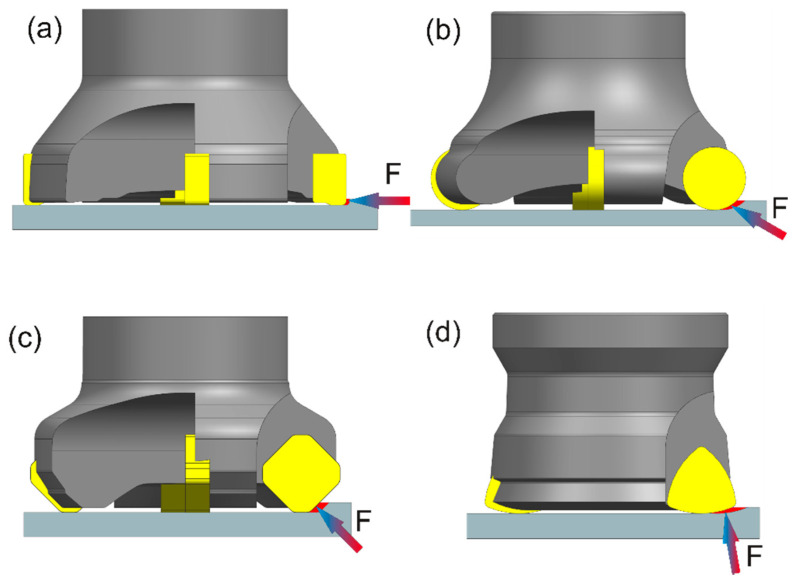
Cutting force directions for various face milling cutter types: (**a**) entering angle of 90°, (**b**) round insert, (**c**) entering angle of 45°, (**d**) entering angle of 10°–20°.

Milling constitutes a technique of manufacturing parts, which is often utilized in the machine industry, chiefly: aviation, automotive, aerospace, and tool industry. It is performed with the use of multi-edge tools (mills) with specified cutting edge geometry, whereas the nature of the edge’s work is interrupted [1]. As a result, cutting edges are sequentially in and out of contact with machined material. Depending on the tool design, one may distinguish many variants of milling. Depending on the milling kinematics, there are two types of milling operations: down milling and up milling. On the other hand, in terms of cutting technology, there are respectively: peripheral milling, end milling, and face milling. The selection of a proper milling variant has a significant impact on the cutting forces [2], temperature [3,4], the dynamics of the milling process, chip formation, as well as the quality of a surface after milling [5,6,7]. Figure 1 presents the types of milling along with the direction of a resultant cutting force.

In the case of face milling with an entering angle of 90° (Figure 1a), the direction of the cutting force is radial [8,9]. The stiffness of the tool in the radial direction is low, which entails the necessity of reducing milling efficiency and may pose an increased risk of an occurrence of machining vibrations [10,11]. Figure 1b–d presents the types of face milling with an entering angle below 90°. In the case of milling with the use of a toroidal cutter, the direction of a resultant cutting force depends on the value of an axial depth of cut. However, the tool load is transmitted both in radial and axial directions. In the case in Figure 1c, the load is transmitted in both directions as well. However, the most advantageous cutting force system is presented in Figure 1d. It shows the type of face milling, in which there is a very small entering angle, of about 10°–20°. As a result, almost all of the cutting force occurs in the axial direction. In that direction, the tool is mainly compressed and its stiffness is high. Hence, it is possible to work with a face mill with, for instance, long overhang or with high feeds [12]. Face milling has found many applications in the tool industry, in the machining of injection molds parts, such as dies, punches, and stamping dies, which are often machined from the heat-treated materials with a hardness of above 30HRC.

**Figure 2:**
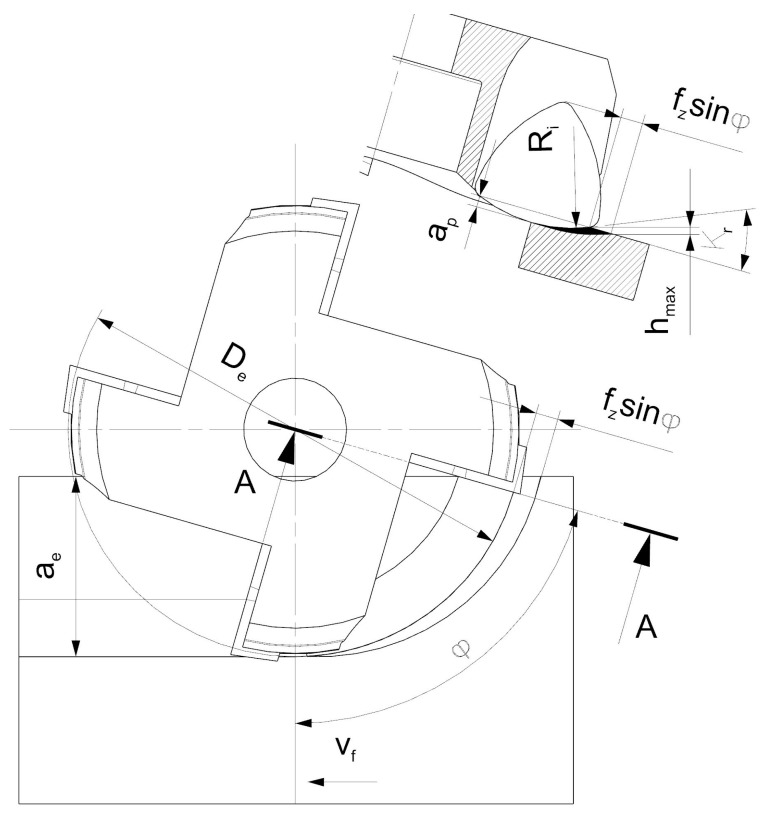
The essence of HFM.

The use of face cutters with a small entering angle has allowed implementation of a new milling method—high feed machining (HFM) [12,13]. The essence of HFM depends on utilizing high values of feed per tooth *f_z_* as well as the radial depth of cut *a_e_*, but low values of axial depth of cut *a_p_*. The *a_p_* parameter is limited by the length of a cutting edge (Figure 2). On the other hand, the design of the tool allows for employing high values of feed per tooth, which in turn results in the increase of the volume of material removed over time. The maximum thickness of the cut layer depends on the engagement angle *φ* and entering angle *κ_r_* as follows:

(1) $$h_{max}\left(\varphi \right)=f_{z}\cdot \sin\varphi \cdot \sin\kappa_{r}$$

The values of feed per tooth may reach up to 4 mm/tooth. Such high values of feed are necessary due to the occurrence of the chip thinning phenomenon as well as to obtain the cut layer thickness greater than the minimal value *h_min_* [14]. These machining conditions can be achieved due to the specific geometry of face cutters, which are characterized by a small entering angle *κ_r_* of about 10°–20°. Despite the significant limitation in axial cutting depth (to about 2 mm), high feed values mean that face milling in HFM technology is regarded as one of the most productive and economical machining methods. An advantageous direction of the resultant cutting force constitutes another significant property of HFM. Small entering angle values ensure that almost all of the cutting force is transmitted in the axial direction of the tool. In the axial direction, the tool is characterized by the highest stiffness, which allows to transmit high loads and to machine a cut layer with a large cross-section [15,16].

The analysis of the literature allows concluding that there are not many works pertaining to the milling process with high feed rates. Most of the works include the analysis of the face milling process with the use of the cutters characterized by conventional cutting insert setup as well as simulation studies of cutting forces during machining of various materials in the face milling process. Li et al. [17] presented a theoretical approach to cutting force modeling during face milling. The model was based on the fundamental knowledge of machining theory. The values of cutting forces were calculated based on the properties of machined material, tool geometry, and machining parameters for machining with the use of a single-edged tool. Moreover, the authors allowed in their cutting force model for cutting insert setup errors, runout errors, and possible machining vibrations of the tool or the workpiece. The model was verified in experimental tests, which allowed comparing the results with the theoretical model. The authors presented a high correlation between the experimental and simulation results. On the other hand, Korkut and Donertas [18] assessed the impact of the variation in cut layer cross-section (feed and depth of cut) and in cutting speed on the values of cutting force components, surface quality, and the possibility of a formation of the built up edge (BUE) during face milling of AISI 1020 and AISI 1040 steel. They concluded that the increase in cutting speeds causes the increase of cutting force components, but with low and moderate cutting speeds, they observed a formation of a built up edge. Sheth and George [19] evaluated the impact of technological parameters, such as rotational speed, feed per tooth, and depth of cut on surface quality and surface flatness during machining of WBC forged steel using ANOVA variance. Fulemova and Janda [20] investigated the influence of cutting edge radius on the values of cutting forces, surface quality, and tool lifetime during face milling. In their tests, the authors used cutting edges manufactured by grinding, broaching, and laser shaping of the edge, in the range of 5–15 µm. The authors concluded that the best results were obtained for the radius of 15 µm, for which the lowest forces were recorded, as well as the lowest surface roughness and the longest tool lifetime. Tapoglou and Anoniadis [21] conducted the simulation tests based on the accurate CAD model of a cutting insert. Consecutive cutting edge positions were generated in order to reproduce the actual kinematics of face milling. The authors predicted the surface roughness and cutting forces values at the simulation stage. The results of the simulation tests were verified in experimental studies during milling of St52-3 and CK60 steel, obtaining a significant correlation between the results. Similar studies were carried out by Munoz-Escalona and Maropoulos [22], with the use of a square cutting insert. The tests were aimed to determine a model for the assessment of surface quality after machining. The model was based on the marks on the machined surface created by cutting inserts. The simulation tests were verified during machining of 7075 aluminum alloy, for varied cutting speeds, feed per tooth, and cutting depths values with the use of two cutting inserts with different radius. The correlation between the experimental and simulation test reached 98%. Karpuschewski et al. [23] developed a strategy for progressive face milling, which allowed for a lower ratio of the width *b* to the thickness *h* of the cutting layer. For this purpose, they developed a new face milling cutter equipped with a system for precise alignment of cutting inserts in order to reduce axial runout. The authors in their studies proved that the decrease of the *b*/*h* ratio has a positive impact on the machining process. It results in lower machining vibrations, which in turn allows increasing the milling performance. Karpuschewski et al. [12] presented simulation studies of the face milling, varying the position of a cutting insert and changing respective rake, relief, and helix angles. The tests were conducted based on the finite element method in the AdvantEdge software (Version 7.4). The authors presented the results of the influence of cutting edge geometry on the cutting force components values and chip shaping process depending on the depth of cut and feed per tooth values.

On the basis of the presented analysis of the literature, one may conclude that the analysis of the high feed face milling process is still an important and discussed issue. However, the available results of the experimental studies pertain to milling using cutters with the entering angle of 90°. The experimental tests focus on the analysis of the influence of rake and relief angles on the milling process. Moreover, there are no works pertaining to surface quality as well as values and distribution of cutting force components during HFM. There is also no research on milling using face cutters with different values of entering angle.

Therefore, in this work, an analysis was focused on two types of face milling tools: a conventional one with *κ_r_* = 45° and a face cutter dedicated for high feed machining. The choice of these cutters is due to the fact that the most commonly used are conventional cutters with the entering angle of 45°. As an alternative, HF cutters with very small entering angle, of about 10°–20°, are used during this study. The change of the entering angle between 45° and 10°–20° is very important, because it affects the shape of the cut layer cross section, as well as the change in the chip forming process. As part of carried out experiment, the research focusing on the influence of face milling parameters and cutting edge geometry on the surface quality, vibration amplitude, and the values of cutting force components was carried out.

## 2. Materials and Methods

**Figure 3:**
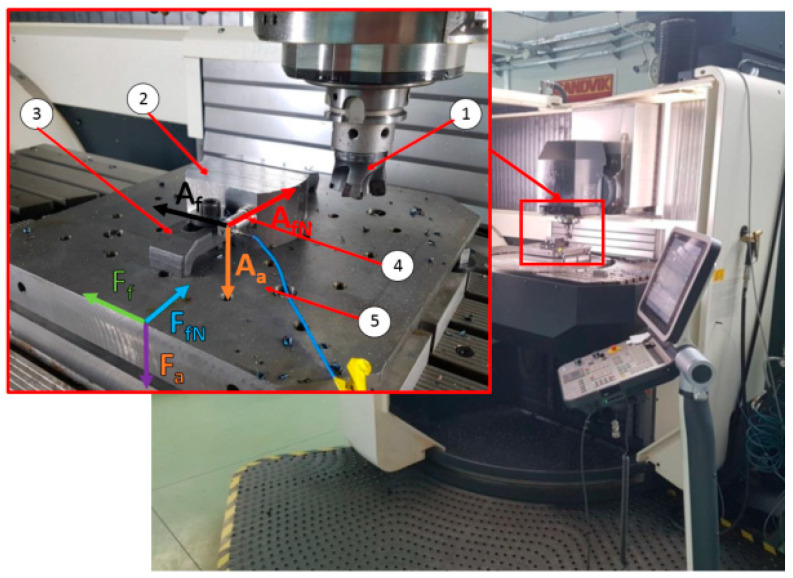
Test stand: 1—HF cutter, 2—milled part, 3—cranked clamp, 4—vibration sensor, 5—cutting force components measuring platform.

The experimental tests were carried out on a DMG’s DMU 100monoBlock multi-axis machining center (DMG, Pleszew, Poland). The machine tool was equipped with specialized measuring equipment dedicated to measuring cutting force components. The measurement of the cutting force components was carried out in the workpiece system. A cutting force measuring platform is an original design consisting of four piezoelectric sensors. Each of these sensors is a three-component Kistler sensor, type 9601A31, Prague, Czech Republic. They are characterized by the measuring range of ±2.5 kN in the direction of the X and Y axes and ±5 kN in the direction of the Z axis. In the milling table plane, *F_x_*(*F_f_*) and *F_y_*(*F_fN_*) cutting force components were measured and in the direction of the Z axis, *F_z_*(*F_a_*) was measured. The voltage signal from the dynamometer was transferred to the National Instruments’ A/D converter type USB-6003, Austin, TX, USA. Moreover, the measurement of the vibration amplitude was measured using a three-component PCB Piezotronic’s vibration sensor type 356B21, Depew, New York, NY, USA. The voltage signal was converted by the National Instruments’ A/D converter type NI-9234. Both signals after conversion were recorded with the use of a Signal Express software. Figure 3 presents the test stand.

The face milling tests were carried out on a 100 mm × 100 mm × 60 mm rectangular 42CrMo4 structural steel test sample with a hardness of 220 HB. The 42CrMo4 steel is commonly used for structural components in machine construction. The face milling tests were conducted for asymmetric up and down milling, with the use of two face milling cutters with the diameter *D_c_* = 63 mm and number of teeth *z* = 4:

- universal face cutter MFA145-063R04A22-SE13 (ENGRAM)—entering angle *κ_r_* = 45°,
- High-Feed face cutter MKB113-063R04A22-SD12 (ENGRAM)—entering angle *κ_r_* = 21.3°.

The evaluation of geometric part specification with the use of surface roughness parameters provides essential information towards the operational and tribological properties of the machined parts [24,25]. Therefore, during this study, the measurement of surface roughness was carried out with the use of a mobile MahrSurf M300 profilometer, Göttingen, Germany equipped with RD18 measuring head, Göttingen, Germany each time after the milling test.

**Figure 4:**
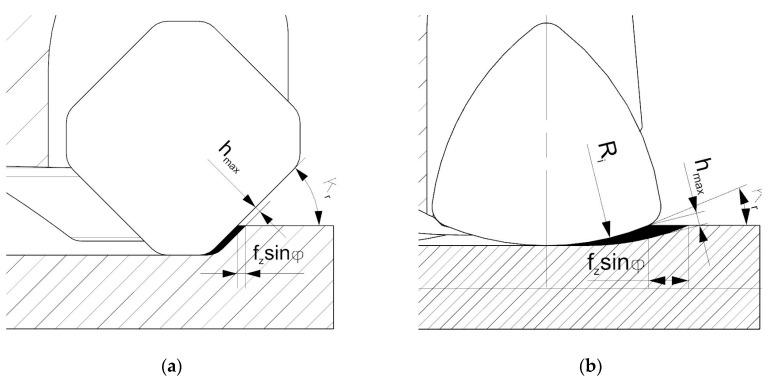
Cut layer cross-section for milling with: (**a**) conventional cutter, (**b**) HF cutter.

The tests were carried out for face milling with varied feed per tooth values. The milling tests were conducted for up and down milling with the use of two different face cutters. The constant parameters were set as follows: cutting speed *v_c_* = 150 m/min, axial depth of cut *a_p_* = 1 mm, and radial depth of cut *a_e_* = 15 mm. The recommended value of *a_p_* for HF cutter is 1–1.2 mm. Thus, the value of *a_p_* = 1 mm was adapted in the tests. The variable parameter was the cut layer thickness *h*, in the range of 0.1–0.25 mm, every 0.5 mm for the engagement angle of *φ* = 90°. Then, due to the asymmetrical milling, the maximum values of *h_max_* for the actual engagement angle of *φ* = 66.4° were determined and were equal to 0.085–0.21 mm (Table 1). The cut layer thickness was varied by changing feed per tooth values. In order to ensure repetitive cutting conditions for both face cutters, feed per tooth values *f_z_* were set for each set cut layer thickness *h* respectively, calculated according to Figure 4.

Moreover, the milling conditions were set as to achieve the same maximal cut layer thickness *h_max_* for both tested cutters. Therefore, in order to assure the same values of *h_max_* during milling with cutters diversified in terms of entering angle, the different sets of feed per tooth values were selected (Table 1). Moreover, a similar cutting volume and the same value of cut layer cross-section area *S_cmax_* = 0.24 mm^2^ were obtained for milling with the use of the conventional face cutter and for milling with the HF cutter with a feed of *f_z_* = 0.28 mm/tooth.

**Figure 5:**
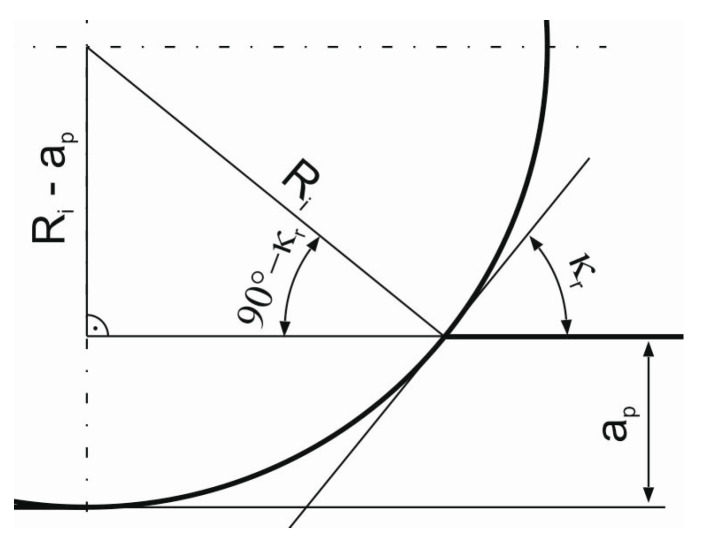
The method of determining the entering angle.

Due to the fact, that in the HF face cutter a cutting edge is a part of a circle, it is necessary to determine a real value of the entering angle. The real value of the entering angle in the HF cutter depends on the *R_i_* radius and the depth of cut *a_p_*. However, with the *a_p_* value, the entering angle can be varied only in a very limited range, i.e., from 5°–20°. The change of the angle in such a range does not affect the cutting process as significantly, because it does not influence the shape of the cut layer cross section and the chip forming process. A method of determining the entering angle is presented in Figure 5. According to Figure 4, the form of the equation allowing to calculate the entering angle for tools with curved cutting edges can be presented as follows:

(2) $$\kappa_{r}=acos\left(\frac{R_{i}-a_{p}}{R_{i}}\right)$$

Based on relationship (2), for the specified axial depth of cut *a_p_* and for the HF cutter, the real entering angle of the cutting insert was determined. The value of the entering angle at a depth of 1 mm was confirmed in a NX CAD system after measuring the cutting insert’s radius *R_i_* on a Mahr’s PRESETTER 2000 tool presetter, Göttingen, Germany. In the case of the HF cutter, the entering angle at a depth of 1 mm was equal to 21.3°. Table 1 presents the determined values of feed per tooth *f_z_* and cutting volumes of removed material for both face cutters used in the experimental tests of face milling.

After each milling test, a surface roughness measurement was carried out with the use of *Ra* and *Rz* parameters, which are commonly used to describe the surface quality of machine parts. *Ra* is the arithmetic mean of the absolute values of the roughness profile ordinates and *Rz* is the arithmetic mean value of the single roughness depth of consecutive sampling lengths. The measurements were conducted in five locations on the test sample in the cutting tool’s feed direction. At the same time, during each experimental test, the cutting force components and vibration amplitude components were recorded as parameters describing the milling process.

## 3. Results and Discussion

### 3.1. The Analysis of Cutting Force Components in HF and Conventional Face Milling

The values of feed *F_f_*, normal to feed *F_fN_*, and axial *F_a_* cutting force components in relation to the variations in cut layer thickness *h_max_*, for up and down milling separately, and for conventional and HF face cutter (different entering angle) were determined. Firstly, the results were presented in the form of box plot graphs.

**Figure 6:**
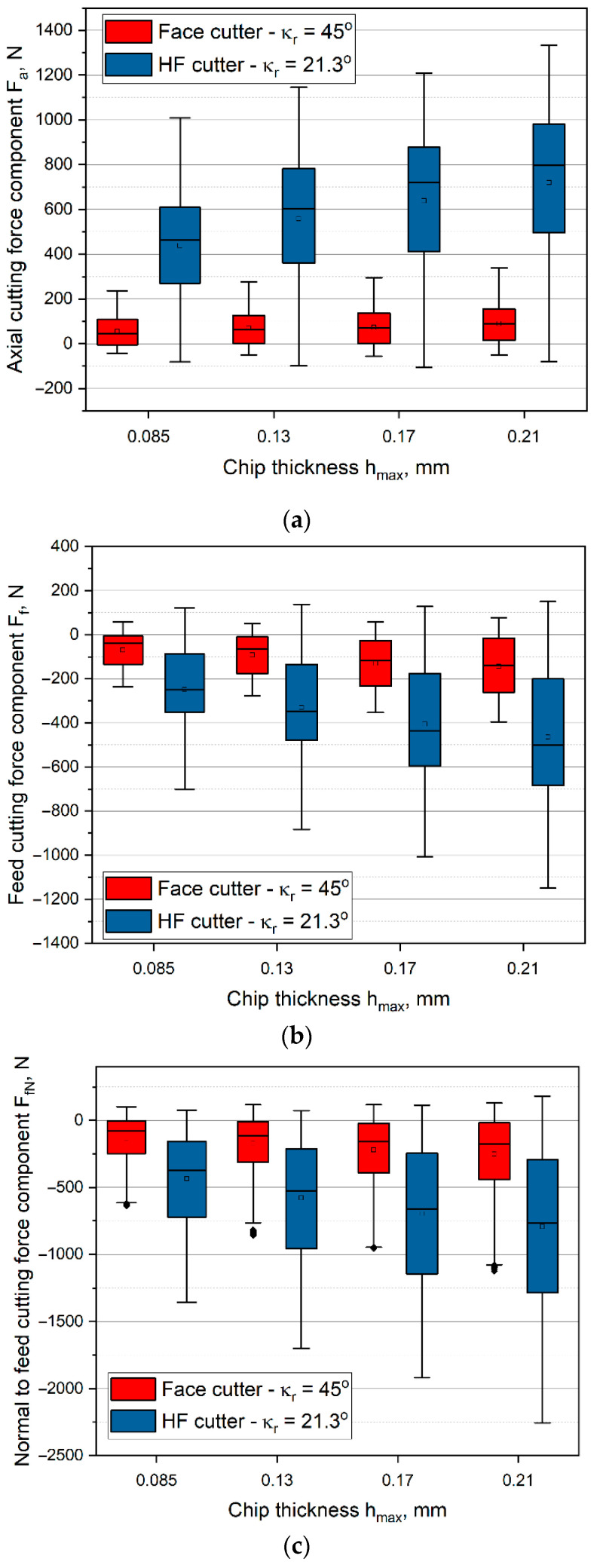
Cutting force components as a function of chip thickness *h_max_* in down milling with *v_c_* = 150 m/min, *f_z_* = 0.14–0.35 mm/tooth for face mill, and *f_z_* = 0.28–0.68 mm/tooth for HF mill: (**a**) *F_a_*, (**b**) *F_f_*, (**c**) *F_fN_*.

Analyzing the box plots presented in Figure 6, for down milling, it can be observed that the increase of cut layer thickness *h_max_* results in a monotonic increase of the mean values of cutting force components, which correlated with the increase of the cut layer cross-sectional area *S_cmax_*. The highest values of cutting force components were recorded for the HF cutter. The experimental results confirm theoretical analyses of milling with the HF cutter, which state that milling with the mentioned tool induces high values of axial cutting force component *F_a_*. Up to eight times increase in axial cutting force component *F_a_* values in relation to the conventional cutter was observed (Figure 6). Moreover, the mean values of the cutting force components in the feed *F_f_* and normal to feed *F_fN_* directions also show a several times increase for the HF cutter in comparison with the conventional cutter (Figure 6). In the case of down milling, the increase of feed cutting force component *F_f_* of about 400% and the increase of normal to feed cutting force component *F_fN_* of about 200% in comparison with the conventional face cutter were observed. A significant increase in all of the cutting force components during milling with the HF cutter can be explained by significantly higher cutting volumes and cut layer cross-sections for the same values of chip thickness *h_max_*.

When analyzing the box plots presented in Figure 6, one can observe notable differences between the range of recorded values and the sizes of boxes. The interquartile range IQR range values were significantly higher for the HF cutter than for the conventional cutter. Thus, the stability of HF milling was lower than that with the conventional cutter. The cutting force components values were characterized by much higher dispersion and were less concentrated around the average value. In addition, for the HF cutter, a much greater symmetry of the cutting force values distribution was observed, in relation to the values reach for a conventional cutter. Furthermore, in case of the measured *F_fN_* component values obtained for the face cutter, the outliers were observed. They indicate that the cutter is less stable in the normal-to-feed direction, which results from the higher value of the entering angle. 

As is presented in Table 1, milling with the HF cutter and chip thickness *h_max_* = 0.085 mm results in the same cutting volume and the same feed of 0.28 mm/tooth as milling with the conventional cutter and chip thickness *h_max_* = 0.17 mm. When comparing the cutting force components for both cutters, it was clear that milling with the HF face cutter results in higher cutting force values. It means, that removing the same volume of the material using an HF face cutter will always result in higher cutting force than when using a conventional cutter. However, the load is transferred mainly in the axial direction. This phenomenon is due to the small entering angle in the HF cutter, which results in a specific cut layer cross-section. A comparison between the values of the cutting force components for both cutters, with the same value of cut layer cross-sectional area, has also been made. A constant value of the cross-sectional area was obtained for milling with the feed of 0.28 mm/tooth. The presented data prove that for the same cut layer cross-sectional area, significantly higher cutting forces occur when machining with the HF cutter. Components *F_f_* and *F_fN_* were about 2 times higher in comparison with milling with the conventional cutter, when *F_a_* component was about 5 times higher. This phenomenon may be explained by the significant difference in the cut layer cross-section shape as well as by the difference in the entering angle. In the case of the HF cutter, most of the load is transmitted in the axial direction.

**Figure 7:**
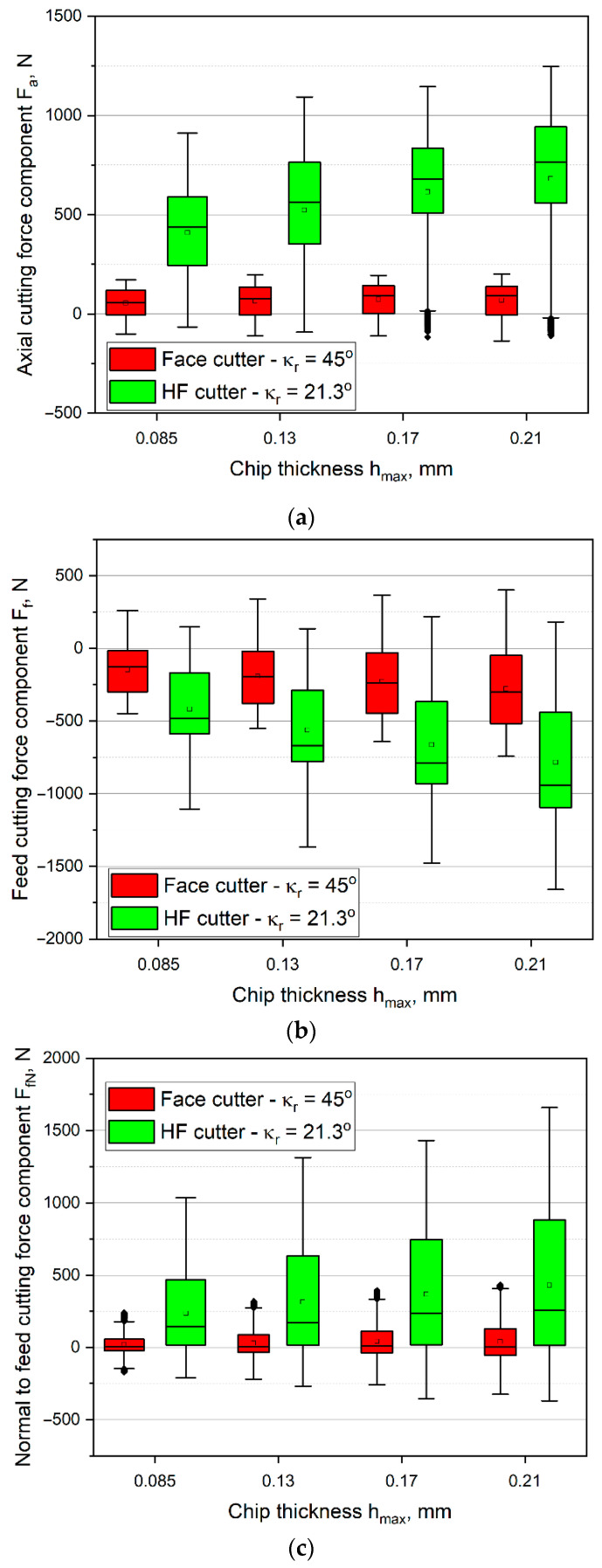
Cutting force components as a function of chip thickness in up milling with *v_c_* = 150 m/min, *f_z_* = 0.14–0.35 mm/tooth for face mill, and *f_z_* = 0.28–0.68 mm/tooth for HF mill: (**a**) *F_a_*, (**b**) *F_f_*, (**c**) *F_fN_*.

Then, the corresponding analyses of up milling were conducted. In Figure 7, the values of cutting force components are presented. Analysis of the results allows observing that adoption of the up milling results in changing the directions of *F_f_* and *F_fN_* cutting force components for the opposite in comparison with down milling. Moreover, in the case of up milling, the recorded mean values of the *F_f_* cutting force component proved to be about 50% higher than in the case of down milling, whereas the recorded mean values of *F_fN_* component were about 50% lower in comparison with down milling. The above-mentioned fact is true for each milling test. As in the case of down milling, also in up milling, machining with the HF cutter was characterized by a much higher dispersion of force values than machining with the conventional cutter. However, a greater asymmetry of the distribution of values in the box can be observed. The median in most cases was not close to the mean value. The relationships between the cutting force component values for both cutters during up and down milling were similar. However, the change in the type of milling did not affect the axial cutting force component values. Subsequently, the values of the cutting force components for up milling with the same feed of 0.28 mm/tooth and the same cut layer cross-sectional area of *S_cmax_* = 0.24 mm^2^ were compared. The *F_a_* component for the HF cutter, analogously as in down milling, increased almost 5 times. For the *F_fN_* component, a 3 times increase was recorded. An increase in *F_f_* component is noteworthy, which in the case of the HF cutter was only approximately 30% in relation to the conventional cutter. This phenomenon results mainly from different mechanisms of material deformation and chip formation occurring during up milling. They are the reason of the increased tool load in the feed direction.

Similarly as with the down milling, for the up milling, a comparison of the cutting force components, for the same cutting volume (for the HF cutter chip thickness of *h* = 0.085 and for the conventional cutter chip thickness of *h* = 0.17 mm), was made. One may observe that for both up and down milling, machining with the HF face cutter resulted in higher cutting force values. The conducted analyses of the cutting force components show that regardless of the milling sample when milling with the use of the HF face cutter, higher values of the axial cutting force component are obtained. However, in the case of other cutting force components, the values and directions depend on the type of milling. Moreover, in case of the highest measured cutting force *F_a_* component values reached for the HF cutter, outliers are present, which indicate a loss of stability of the cutter in the axial direction. Similarly to the down milling experiment, in up milling conducted with the face cutter, outliers within a measured *F_fN_* forces were observed, which results from the cutter lower stability in the normal-to-feed direction.

**Figure 8:**
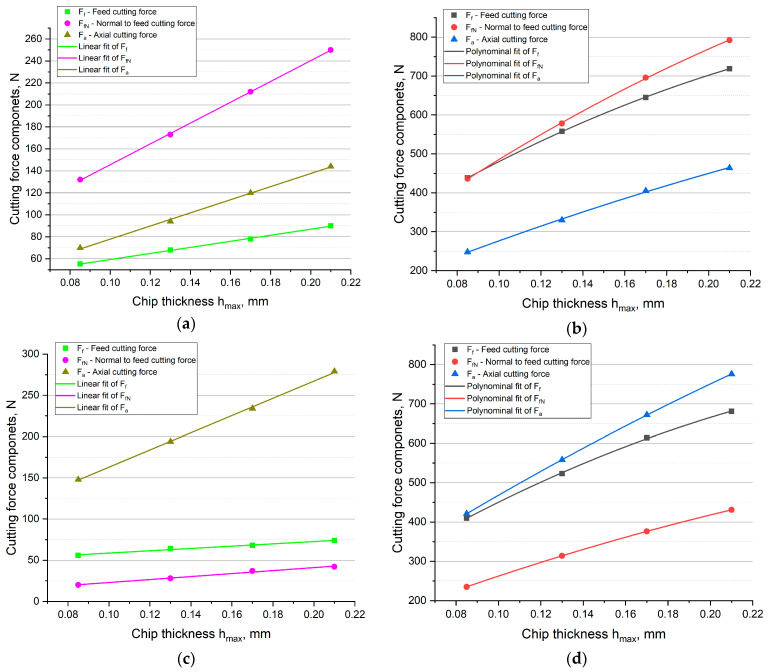
Curve fit for: (**a**) down milling with the conventional cutter, (**b**) down milling with the HF cutter, (**c**) up milling with the conventional cutter, (**d**) up milling with the HF cutter.

Due to the differences between the values of the cutting force components for the conventional cutter and the HF cutter, an attempt was made to determine the relationships describing the variations in cutting force components as a function of chip thickness. To the average values of the cutting force components, the curves were fitted to achieve to highest *R*^2^ coefficient. The results are presented in Figure 8.

The conducted analyses indicate that in the case of machining with the conventional cutter, the highest *R*^2^ coefficient was obtained with linear regression, and in the case of the HF cutter, the best fit was achieved with a non-linear polynomial fit. But in the case of HF cutter, the influence of uncut chip thickness on the cutting force components is close to linear (*R*^2^ = 0.97), therefore the application of a simple regression equation in a form of linear function may be enough for the reliable characterization of force variation as a function of uncut chip thickness. However, the highest *R*^2^ value was obtained for the non-linear polynomial fit. In the case of down milling, for the conventional cutter, the following equations were calculated:

(3) $$y=31.79+275.68h_{max},\;R^{2}=0.998\;y=50.94+946.63h_{max},\;R^{2}=0.999\;y=18.2+596.95h_{max},\;R^{2}=0.997,$$

and for the HF cutter the equations can be written as follows:

(4) $$y=158.61+3713.55h-4983.15h_{max}^{2},\;R^{2}=0.999\;y=112.09+4184.74h-4492.01h_{max}^{2},\;R^{2}=0.999\;y=63.44+2331.01h-1994.5h_{max}^{2},\;R^{2}=0.998.$$

The same types of equations were obtained for the up milling. For the conventional cutter the relationships were formulated:

(5) $$y=44.66+140.1h_{max},\;R^{2}=0.984\;y=4.85+180.86h_{max},\;R^{2}=0.985\;y=58.61+1042.93h_{max},\;R^{2}=0.999,$$

and for the HF cutter the equations were as follows:

(6) $$y=135.17+3647.92h-4975.03h_{max}^{2},\;R^{2}=0.999\;y=60.56+2249.12h-2310.91h_{max}^{2},\;R^{2}=1\;y=131.5+3631.96h-2677.23h_{max}^{2},\;R^{2}=1.$$

In all the cases, the fit of the model was very good because the coefficients of determination were equal *R*^2^ = 0.984–1.

The conducted analyses indicate that the shape of the cutting edge and the entering angle influence not only the values of the cutting force components but their functional relationship as well. In the case of the face milling with the conventional cutter, the cutting force components were linearly dependent on the thickness of the cut layer. It means that the forces increased proportionally with increasing the cut layer cross-section. In the case of the HF cutter, the relationship was non-linear. The best fit was achieved for the second degree polynomial. The equations and curves indicate that with the increase of the cut layer thickness, the forces increase, but non-linearly. This phenomenon may be explained by the fact, that the cut layer cross-sectional area of the HF cutter varies significantly from the regular area approximated by a parallelogram. Moreover, when milling with the HF cutter, the cross-sectional area varies alongside the variations in thickness *h_max_*. With the increase in *h_max_*, the share of plastic deformations and cold-work hardening is lowered. As a consequence, an increase in the thickness *h* increases the share of machining (decohesion of the material) in relation to the plastic deformation and burnishing. On the other hand, with lower values of the cut layer thickness *h* close to *h_min_*, the elastic-plastic effects on the cutting edge increase. These phenomena indicate that in the case of the HF cutter, the cutting force components are described by different relationships than in conventional milling.

### 3.2. Analysis of the Vibration Amplitude Components during HF and Conventional Face Milling

**Figure 9:**
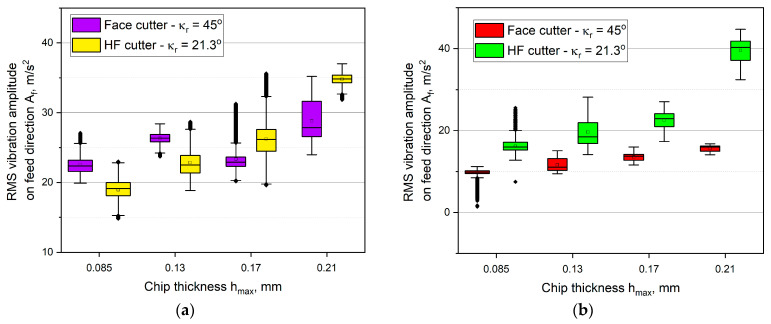
The influence of the chip thickness on the vibration amplitude values in the feed direction *A_f_*: (**a**) up milling, (**b**) down milling.

**Figure 10:**
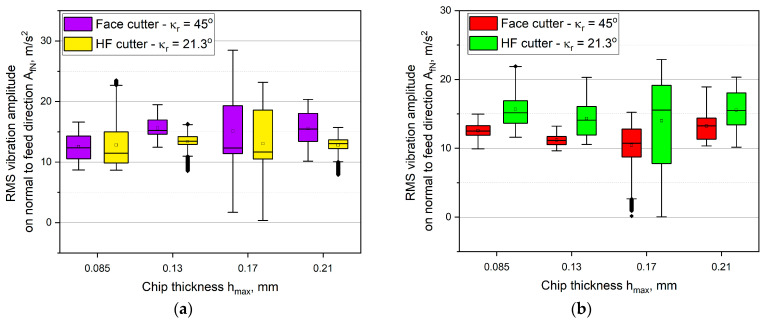
The influence of the chip thickness on the vibration amplitude values in the normal to feed direction *A_fN_*: (**a**) up milling, (**b**) down milling.

**Figure 11:**
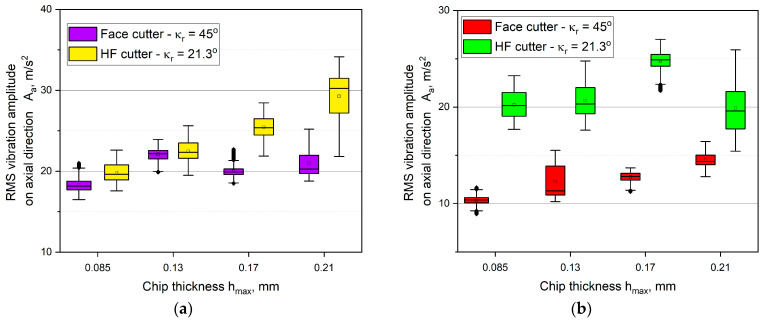
The influence of the chip thickness on the vibration amplitude values in the axial direction *A_a_*: (**a**) up milling, (**b**) down milling.

In Figure 9, Figure 10 and Figure 11, the RMS values of the vibration amplitude components recorded during HF and conventional, up and down milling in relation to the thickness of the cut layer are presented. Analyzing the obtained results of the vibration amplitude in the feed direction *A_f_* it can be observed, that up milling and down milling are significantly different. In the case of down milling, machining with the HF cutter induced higher vibration amplitudes than with the conventional cutter. Moreover, the highest values of the vibration amplitude were recorded during down milling with the use of the HF cutter (Figure 9). The maximum value reached about 40 m/s^2^ for the chip thickness of *h_max_* = 0.21 mm. It was also observed that the vibrations during conventional milling were characterized by lower dispersion (lower size of the box and the whiskers). Only the down milling with both of the cutters was very unstable, which is described by the high number of outliers. On the other hand, for up milling, an average of about 7% decrease in comparison with all of the down milling tests was obtained. The highest decrease in vibration amplitude during up milling was noted for the thickness of *h_max_* = 0.21 mm and it was equal to approximately 19%, in comparison with the HF cutter down milling. One may also observe that the up milling process was characterized by the lack of dependence between the vibration amplitude, thickness *h_max_*, and the type of the cutter. The range of vibration amplitude was high, and the average values are alternately higher for the HF cutter or the conventional cutter. However, the lowest vibrations were obtained for down milling with the conventional cutter, which did not exceed 15 m/s^2^ for all of the tested chip thicknesses *h_max_*. It was also observed that lower vibration amplitude values accompany the lower values of the feed cutting force component *F_f_* (Figure 6, Figure 7 and Figure 9). In almost every milling test, numerous outliers were observed, which indicates lower stability in the feed direction in comparison with down milling.

Figure 10 presents the influence of the chip thickness on the vibration amplitude variations in the normal to feed direction *A_fN_*. It has been noticed that the changes were of an irregular and non-monotonic form as the chip thickness *h_max_* increases. It can also be observed that in down milling, the lowest values were recorded for the conventional cutter, but in up milling, the lowest values were recorded for the HF cutter. It was also noted that for the chip thickness of *h* = 0.17 mm, the milling process was highly unstable, which can be inferred from long whiskers or numerous outliers. The highest statistical dispersion and the largest boxes were obtained. Furthermore, in the case of up milling, the distributions of the signal were very asymmetrical. In case of the up milling, for the HF cutter, numerous outliers were observed as well, which indicates a significant dispersion of vibration amplitude.

In case of the axial vibration amplitude component *A_a_*, regardless of the type of milling and the thickness *h_max_*, the higher values were noted for the HF cutter than for the conventional cutter. The values of the vibration amplitude signal were equal on average to approximately 25 m/s^2^ for down milling and to 20 m/s^2^ for up milling. The lowest vibrations were recorded for the conventional face cutter and they did not exceed 13 m/s^2^. The sizes of the boxes were significantly lower than for the other components. It is clear that the component *A_a_* was dependent on the thickness *h_max_*. In addition, in some cases, minor outliers were observed. However, their presence is accompanied by boxes of a very small size. Small boxes indicate high skewness of the distribution, thus the number of the outliers is of lower importance.

The presented graphs lead to a conclusion that the force components system induced during milling with the use of the HF cutter leads to significant vibration amplitudes, which may result in the lack of milling stability, cause accelerated tool wear, and have a negative impact on the machined surface quality. Another reason for obtaining higher vibration amplitudes may be due to the chip forming process. The HF cutter is characterized by a longer active cutting edge (Figure 2) than a conventional one. Moreover, the low value of the entering angle may contribute to the higher plastic deformation of the material and its strain hardening instead of shearing. This, in turn, results in higher vibrations.

### 3.3. Analysis of the Surface Roughness after HF and Conventional Face Milling

**Figure 12:**
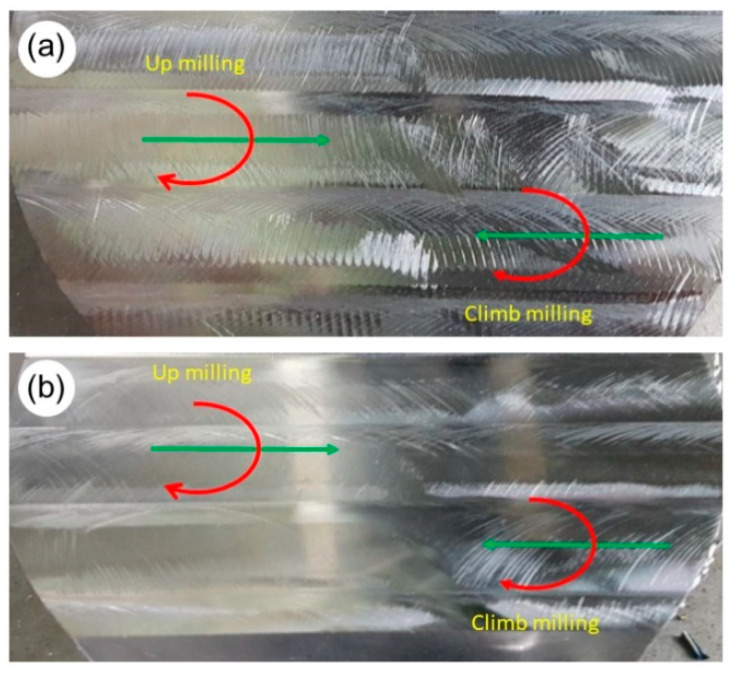
Surface integrity after milling using: (**a**) HF face cutter, (**b**) face cutter with the entering angle of κ*_r_* = 45°.

Figure 12 presents the surface integrity after up and down milling with the use of the HF cutter as well as the conventional face cutter with the entering angle of *κ_r_* = 45°.

Assessment of the surface integrity allows to state unequivocally that the surface after machining with the HF cutter contains numerous imperfections in the form of clearly visible unevenness both after up and down milling (Figure 12a). The effect of that surface integrity presents itself in the form of an increase in the axial component of the cutting force in comparison with the conventional cutter as shown in Figure 8. Higher values of the axial force result in generating higher vibration amplitudes (see Figure 9). This in turn can cause greater axial oscillation of the cutting edge, which affects the integrity of the surface after milling. Moreover, the machine tool that was a part of the test stand is equipped with an extendable Y axis, which may result in a lower stiffness of the machine tool—tool—workpiece system. This, in turn, may contribute to excessive vibrations during machining with the HF cutter, which is more heavily loaded in the axial direction, despite using as small an overhang as possible during tests. On the other hand, Figure 12b proves that milling with the use of the conventional face cutter with the entering angle of *κ_r_* = 45° allows obtaining significantly better surface integrity than milling with the HF cutter.

**Figure 13:**
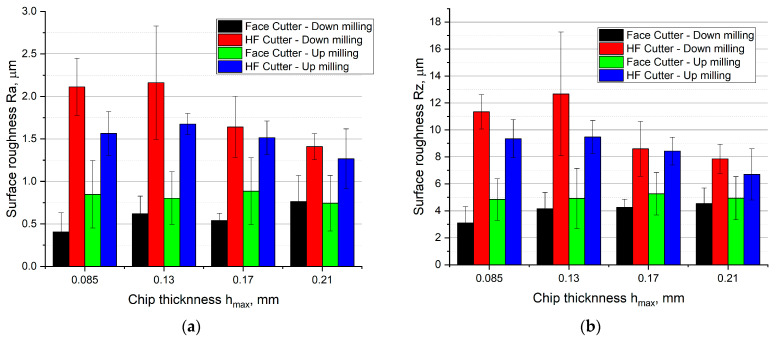
The influence of chip thickness on the surface roughness (**a**) *Ra* and (**b**) *Rz* after up and down milling with the HF and conventional cutter.

Figure 13 presents the results of surface roughness measurement in the form of *Ra* and *Rz* parameters after up and down milling with the use of the HF cutter and the conventional face cutter as a function of cut layer thickness *h* variations.

Analysis of the results allows to state that using the HF cutter results in a significant increase in surface roughness in comparison with the conventional cutter with the entering angle of 45° during both up and down milling. An average relative increase of approximately 240% in *Ra* parameter and of approximately 160% in *Rz* parameter was noted during down milling. For up milling, an average relative increase of approximately 70% in *Ra* and *Rz* parameters in relation to the cutter with the entering angle of *κ_r_* = 45° was noted. The highest relative increase was observed for low cut layer thickness *h_max_*. The relative increase in *Ra* and *Rz* roughness parameters in the case of *h_max_* = 0.085 mm for down milling was equal to approximately 420% for *Ra* parameter and approximately 161% for *Rz* parameter. However, for up milling, the increase was equal to approximately 84% for *Ra* parameter and approximately 93% for *Rz* parameter, all in comparison with the conventional face cutter.

It can also be noted that an increase in cut layer thickness causes a decrease of *Ra* and *Rz* surface roughness in the case of the HF cutter, but in the case of the face cutter with the entering angle of *κ_r_* = 45° a small monotonic increase in surface roughness parameters is observed. As a result, for the chip thickness of *h_max_* = 0.21 mm, in the case of the HF cutter in comparison with the conventional face cutter, a relative increase of approximately 85% in *Ra* parameter and of approximately 73% in *Rz* parameter for down milling as well as a relative increase of approximately 70% in *Ra* parameter and of approximately 35% in *Rz* parameter for up milling were observed.

**Figure 14:**
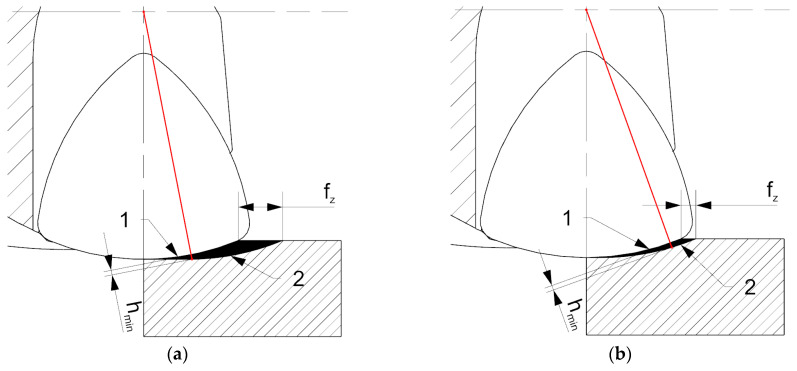
The influence of feed rate on cutting zones in HF milling: (**a**) high feed rate, (**b**) low feed rate. 1—elastic-plastic zone, 2—cutting zone (decohesion of the material).

A decrease in surface roughness parameters in the case of the HF cutter and higher cut layer thicknesses *h* (feed per tooth *f_z_*) can be explained by the reduction in the effect of chip thinning resulting from the geometrical setup and the design of a cutting insert (Figure 14).

An increase in feed per tooth and following cut layer thickness cause, in the case of the HF cutter, a reduction of the area of cutting layer with a thickness below the minimal cut layer thickness *h_min_* formed along a cutting edge (Figure 14a). Due to that fact, the increase in cut layer thickness *h* causes an increase in the cutting process (decohesion of the material) in relation to the plastic deformation and burnishing. On the other hand, lower values of cut layer thickness *h* (feed per tooth *f_z_*) cause an increase of the elastic-plastic process of the cutting edge in relation to the cutting (decohesion of the material). Increasing the share of plastic deformation and burnishing causes deterioration of surface quality (Figure 14b). This phenomenon does not occur in the case of a conventional face cutter due to the higher entering angle as well as shorter active cutting edge involved in the machining process [26,27].

## 4. Conclusions

Face milling constitutes one of the main types of the milling process, which is widely used in many industrial applications. Modern tools, numerically controlled machine tools with high stiffness and CAM software allowed for the development of modern milling methods, which include HF milling. Therefore, experimental tests were carried out, aimed at comparing the face milling process with the use of a conventional face cutter with the entering angle of *κ_r_* = 45° as well as the HF cutter dedicated for milling with high feed rates. The distribution of the cutting force components, vibration amplitude, and surface integrity were analyzed. Research shows that:

- using the HF cutter, it is possible to achieve over twice the cutting volumes obtained during milling with the use of a conventional face cutter with the entering angle of *κ_r_* = 45°, with constant cut layer thickness *h_max_*;
- during milling with the HF cutter, higher values of the cutting force components occur, eight times the increase in the value of the axial cutting force component *F_a_*, four times the increase in the feed cutting force component *F_f_*, and two times the increase in the normal to feed component *F_fN_* were recorded;
- despite the different shape of the cut layer cross-section during milling with the HF cutter, specific values of the cutting force in relation to the cut layer cross-section are similar for both tool;
- taking into consideration the distribution and values of the cutting force components (mainly high values of the axial component, exerting load on spindle’s bearings) as well as vibration amplitude values, it can be concluded, that the HF milling process should take place on stiff machine tools, of which the design enables transferring high loads in the direction of a spindle’s axis;
- milling with the use of the HF cutter in most cases contributes to occurring higher vibration amplitudes, compared with milling with the face cutter with the entering angle of *κ_r_* = 45°, which results from different values of cut layer thickness in cross-sectional area;
- the reason for the higher level of vibrations is the chip forming process as well as the shape and size of a cut layer during milling with the HF cutter, where cut layer thickness *h* varies with the depth of cut and where there is an area below *h_min_*, where elastic-plastic deformation of the material is increased and the tool is deflected;
- the average vibration RMS values in the feed and normal-to-feed directions for up milling with the HF cutter are equal to or even lower than for the conventional cutter. It means that in some machining conditions, milling with the HF cutter presents an alternative for the conventional cutter, with significantly higher machining efficiency and similar machining loads;
- the use of the HF cutter causes significantly higher surface roughness, in comparison with the conventional face cutter with the entering angle of *κ_r_* = 45°, due to the variation in cut layer cross-sectional thickness and increase in plastic deformation of the material;
- the increase in cut layer thickness during HF milling results in reducing the surface roughness, which can be explained by the reduction of the elastic-plastic area in the cut layer below the value of the minimal cut layer *h_min_*.

## Figures and Tables

**Table 1:** The range of tested parameters.

Chip thickness *h_max_*, mm	0.085	0.13	0.17	0.21	Type of mill cutter
Cross section area *S_cmax_*, mm^2^	0.12	0.18	0.24	0.3	Face mill
	0.24	0.35	0.45	0.55	HF face mill
Feed per tooth *f_z_*, mm/tooth	0.14	0.21	0.28	0.35	Face mill
	0.28	0.42	0.55	0.68	HF face mill
Cutting efficiency *Q_w_*, mm^3^/min	6417	9815	12835	15855	Face mill
	12,472	19,106	24,958	30,854	HF face mill

## Data Availability

The data presented in this study are available on request from the corresponding author.
